# A Survey of* Gopherus polyphemus* Intestinal Parasites in South Florida

**DOI:** 10.1155/2018/3048795

**Published:** 2018-12-26

**Authors:** Jessica N. Huffman, Kent S. Haizlett, Dana K. Elhassani, Brian T. Cooney, Evelyn M. Frazier

**Affiliations:** Department of Biological Science, Florida Atlantic University, Boca Raton, FL 33431, USA

## Abstract

*Gopherus polyphemus* populations are diminishing throughout their range due to urbanization, fragmentation, and poor habitat management. Increased population densities, poor habitat quality, and lack of fire may influence disease transmission. Parasite roles within wild tortoise populations are largely unknown; despite evidence these pathogens may pose significant health risks. This study provides a baseline of gopher tortoise intestinal parasites across South Florida and reports on how varying environmental and tortoise characteristics may affect intestinal parasite species prevalence and approximate loads. Tortoise fecal samples were taken from six tortoise populations across five South Florida sites. Seven species of intestinal parasites were discovered from 123 tortoises. Identified parasites include endohelminths such as cyathostomes, pinworms, ascarids, flukes, and protozoans including* Eimeria*,* Cryptosporidium*, and* Amoeba* species. Significant differences in parasite prevalence and loads were seen between sites, while parasitism among sex, size class, and habitat type remained relatively ubiquitous.

## 1. Introduction

The gopher tortoise (*Gopherus polyphemus*) is endemic to the southeastern region of the United States [1]. This chelonian generally resides in dry, sandy soil environments [2, 3] and is found in a diverse range of ecosystems (inland, coastal, and island) [3]. These medium-sized tortoises construct extensive burrows that provide shelter and protection to more than 350 vertebrate and invertebrate species [4], many of which are also protected by federal and state law [5]. Due to the benefits that they provide,* G. polyphemus* are recognized as a keystone species, directly affecting the biodiversity and health of the ecosystems they inhabit [6].

Unfortunately, gopher tortoise populations have experienced rapid declines throughout their native range [3]. As a result,* G. polyphemus* populations west of the Mobile and Tombigbee Rivers in Alabama are federally threatened under the U.S. Endangered Species Act and those in their eastern range are classified as a candidate for federal listing as Threatened [7]. Over the past century, Florida's gopher tortoise populations have declined over 80% [3, 8, 9]. Agricultural modifications of natural environments and the rise of urbanization have resulted in habitat loss and fragmentation [10]. These fragments are often surrounded by development [3, 11] which can restrict the space available for populations to disperse [12], as well as limiting food availability. Because of limited migration capabilities, accompanied by decreased habitat size, populations may become overcrowded. Crowding may lead to a variety of negative health consequences including higher infection rates, as well as increased disease transmission and susceptibility [13–16].

Prior to human development, fire played an important role in naturally maintaining gopher tortoise habitats [12, 17]. Ever-increasing urbanization has led to fire suppression [12, 18]. Fire has been shown to reduce canopy cover [19, 20] and control various parasites such as ticks [21]. Areas lacking fire will undoubtedly have more vegetation cover which can limit low herbaceous growth [22–24], allowing less sunlight to reach the ground. This is an important consideration, as ultraviolet radiation has been shown to be effective against various protozoal and bacterial cysts, nematode eggs (affecting some more than others), and most larvae [25].

Recently, the role of pathogens has garnered more attention in conservation biology [26] with growing evidence that infections and pathogens pose an increased health risk as habitats shrink and host populations become more concentrated [27]. However, the effect of disease and parasites on gopher tortoise populations is poorly understood. To date, there have only been six studies investigating the intestinal parasites of approximately 159 gopher tortoises [28–33]. Identified parasites in these surveys included pinworms (*Alaeuris* spp. and* Tachygonetria *spp.), capillarids, ascarids, strongyles (*Chapiniella* spp.), an unidentified trichostrongyle, cestodes (*Oochoristica* spp.), an acanthocephalan (*Neoechinorhynchus pseudemydis*), and the apicomplexan protists* Eimeria paynei*, and* Cryptosporidium, *and possibly* Entamoeba *spp. [28–33].

Unfortunately, these previous investigations have only focused their efforts on identifying* G. polyphemus* intestinal parasites in more northern, temperate regions (Georgia and Louisiana), apart from the Oxyurid species identified in tortoises from Lake Placid, Florida by Petter and Douglass (1976) [30]. Therefore, it is still unclear if gopher tortoise populations in the more subtropical regions of their range (South Florida) will harbor the same endoparasite species and have the same prevalence, as other surveys. Information on the geographical distribution of parasites is considered important, as rising global temperatures are causing shifts in species geographic distributions and it is unclear how populations and their parasitic symbionts may react. Confounding this, numerous studies have projected that increasing temperatures can intensify some disease incidences [34–36] and that climatic aspects (i.e., weather, temperature, precipitation, etc.) are the important influencers of disease transmission [37].

The objectives of this study were to establish a baseline of gastrointestinal parasites in South Florida and evaluate how varying tortoise (host) and environmental characteristics may affect* Gopherus polyphemus* endoparasite species, prevalence, and approximate loads. Four small, urban sites in Palm Beach County and Indian River County and a larger, more natural site in Martin County were selected for this study. These sites vary in size, habitat types, and management regimes, among other population and environmental characteristics. We hypothesized that* G. polyphemus* intestinal parasite prevalence and loads would differ between varying (1) host (sex, size class) and (2) environmental (site, habitat type) characteristics.

## 2. Material and Methods

### 2.1. Study Sites

**Figure 1:**
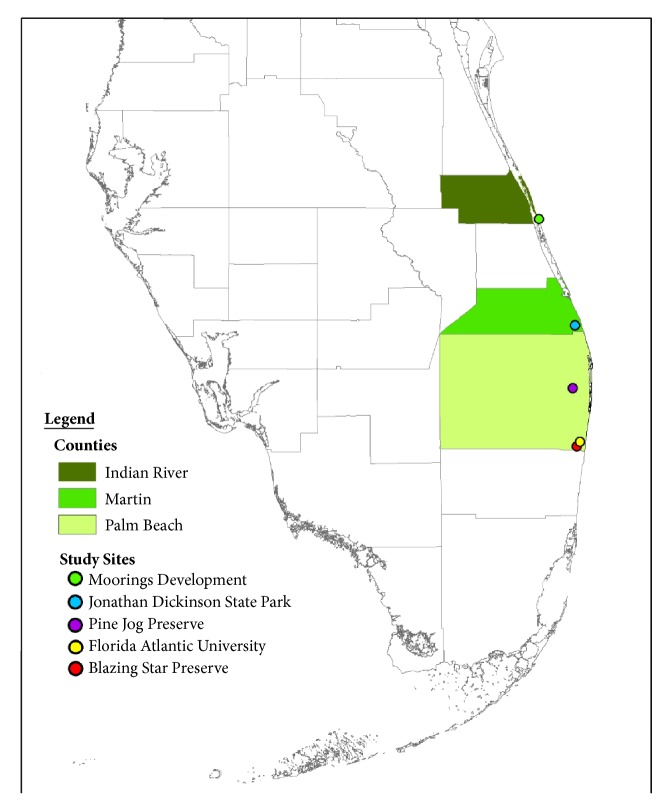
Locations of the five study sites in Florida, USA, from which the six gopher tortoise populations were sampled. Jonathan Dickinson State Park contained two of the study populations, one in Pineland and the other in Scrub.

*Gopherus polyphemus* fecal samples were obtained from six tortoise populations across five South Florida sites (Figure 1): (1) Blazing Star Preserve (BSP), (2) Florida Atlantic University Preserve (FAUP), (3) Pine Jog Preserve (PJP), (4) Jonathan Dickinson State Park (JDSP), and (5) Moorings Development (MD). Jonathan Dickinson State Park contained two of the study populations, one in Pineland (JDSP Pine) and the other in Scrub (JDSP Scrub). All sites are in the subtropical zone (latitudes 23.5° through 35° North).

(1) BSP (26.3473° North, 80.1194° West) is in Palm Beach County and consists of a 10.93-hectare scrubland overridden by invasive plants and overgrown native flora (i.e., sand pine scrub forest, saw palmetto, sand live oak, myrtle oak, and chapman oak). This area currently undergoes both mechanical and chemical management practices. (2) FAUP (26.3712° North, 80.1017° West), located in Palm Beach County on the FAU-Boca Raton campus, covers 36.83 hectares and is divided into two habitat fragments [38]. The larger, western fragment is mostly composed of xeric oak scrub mixed with patches of oak hammock and saw palmetto, multiple invasive species, and mowed grassy triangle to the North. The smaller, eastern portion consists of regularly mowed grasses (native and non-native). These two fragments are considered one, as frequent tortoise crossings are often observed [38]. The current management plan includes mechanical management/bush hogging and herbicide treatments. (3) PJP (26.6648° North, 80.1412° West), located in Palm Beach County, is 54.63 hectares of primarily pine flatwoods habitat, with the remaining 15 acres consisting of prairie, wetland, and oak hammock. This area has historically undergone intensive mechanical and chemical management with the recent implementation of limited prescribed fire beginning in 2009. (4) JDSP (27.0061° North, 80.1289° West), located in Martin County, is a 4,653.88 hectare, rigorously maintained habitat (mechanical, herbicidal, and regular prescribed fire). The park contains 16 distinct natural communities; however, areas suitable for gopher tortoise habitation include mesic flatwoods, sandhill, scrub, and scrubby flatwoods. Since JDSP is the largest, most appropriately maintained site and includes two of the habitat types (pine flatwoods and scrub) that three of our sites (BSP, FAUP, and PJP) are comprised of, it will be used for comparison throughout this study. As aforementioned, two of our sample populations were taken from JDSP: one from scrub and one from pine. JDSP Scrub consists of approximately 1,214.06 hectares, while JDSP Pine consists of approximately 2,428.11 hectares. (5) MD (27.583555° North and -80.328601° West) is located in the South Beach community on North Hutchinson Island (Orchid Island) in Indian River County, FL. The site is approximately 1.38 hectares of remnant beach dune and coastal strand habitat [39, 40] fitted just west of the Atlantic Ocean.

Each of these sites had previous population surveys conducted; however, the methods of data collection, as well as the survey year, varied. BSP and FAUP were surveyed using Belt Transects in 2011 [38, J. Scholl, unpublished data]. PJP was surveyed in 2017 also utilizing Belt Transects [N. Frendberg, unpublished data]. After applying the Florida Fish & Wildlife's correction factor of dividing the number of active burrows by two (FWC, 2008), the estimated populations at BSP, FAUP, and PJP were approximately 2.20, 2.73, and 0.95 tortoises per hectare, respectively. JDSP was surveyed in 2015 using Line Transect Distance Surveys by the Joseph W. Jones Ecological Research Center under a contract with FWC. Using the methods outlined in the 2009 Gopher Tortoise Survey Handbook [41], they estimated JDSP's population to be approximately 0.769 tortoises per hectare (FWC, unpublished data). MD was surveyed most recently in 2017 during a total population survey where 47 individuals were counted (J. Moore, unpublished data) providing an approximate density of 34.05 tortoises per hectare.

The difficulties in accurately comparing population density data acquired from different survey methods should be acknowledged. In fact, abundance estimates via transect surveys are generally larger for the same area compared to complete surveys [42] and the use of broad correction factors to estimate population is cautioned [19, 43, 44] since tortoise occupancy per burrow is known to vary in respect to time and space [45–47]. Although discrepancies exist, we felt that providing population density estimates utilizing these differing methodologies would still offer a rough approximation of density and be useful in terms of general site description.

### 2.2. Research Design and Analysis

Gopher tortoises were hand-captured during field surveys conducted from 2013 to 2017. Fresh fecal matter was collected and placed into 5 mL collection tubes containing SAF (sodium acetate, acetic acid, formaldehyde, and distilled water) in a 3:1 (SAF: feces) ratio [48]. Tortoise carapace length was used to determine size class (adult, sub adult, and juvenile) [49, 50] and sex determination was based on shell morphology [51]. To prevent resampling the same tortoises as a new individual, each tortoise was marked on their scutes utilizing a preestablished numbering system [12]. Once sampling was complete, tortoises were released relative to their location of capture. Equipment used was wiped down with 0.05% chlorohexidine solution to prevent infections between tortoises. Fecal samples were submitted to National Bio Vet Laboratory® in Miami, Florida, for parasitological examination and intestinal parasite identification. Endoparasite identification included multiple methods: (1) fecal flotations, (2) sedimentation, (3) trichrome staining, and (4) Direct Fluorescent Antibody tests (DFA) for* Cryptosporidium.*

The identification data acquired from National Bio Vet Laboratory® was used to determine the prevalence of intestinal parasites. Prevalence refers to the proportion of infected gopher tortoise individuals among all individuals sampled [52]. National Bio Vet Laboratory® also provided estimates of egg and oocyst counts during identification. These counts were approximated by taking the average number eggs/oocysts per high power and low power fields of view (HPF and LPF, respectively) when samples were examined under a light microscope and separated into four load categories (– (0/HPF), + (<1-1/LPF), ++ (2-4/LPF), and +++ (>=5/LPF)). These load categories are simply reference intervals in which no clinical inference can be made. It should also be noted that egg counts are estimated from the number of eggs shedding during a single sampling period and are not reflective of true infection intensities or the number of the adult worms in the gut. From this information, we assigned approximate qualitative load descriptions; none, low, moderate, and high, respectively. Again, these qualitative assignments are not derived from a literature reference and therefore host health effects or degree of parasitism cannot be inferred from these descriptions.

Two-sided Fisher's Exact Tests were completed to determine if the proportions of gopher tortoises infected by the different parasite species (prevalence) would differ between sexes, size classes, sites, and habitat types. A ranked Mann Whitney U analysis was conducted to determine if there were differences in the approximate parasite loads between sexes. Ranked Kruskal-Wallis analyses were completed to determine if there were differences in the approximate parasite loads between size classes, sites, and habitat types. 112 unique fecal samples were used in the analyses which included only known adult (n=104) and subadult (n=8) tortoises due to the low sample size of juveniles (n=3). Therefore, tortoises of unknown size classes (n=8) were also excluded from the analyses.

## 3. Results

A total of 126 fecal samples were collected from 123 tortoises from 2013 through 2017. Three tortoises had feces collected twice when encountered more than once during field surveys. Two of these resampled tortoises (A and B) were from FAUP, and one (C) was from BSP. Tortoise A was an adult male and was captured in October 2014 and then again in June 2015. Tortoise B was an adult female captured in December 2013 and then again in June 2016. Tortoise C was an adult female captured twice in 2015 (June and July).

Seven types of endoparasites were observed in this study. Parasites were identified to genus and include multiple endohelminths such as cyathostomes (*Chapiniella)*, pinworms (*Tachygonetria*), ascarids (*Augusticaecum*), flukes (*Telorchis*), and protozoans including* Eimeria* and* Cryptosporidium*, as well as different amoeba species. Endoparasites were not described to species since definitive identification entails retrieving adult worms residing in the gut, which could not be done unless a deceased tortoise was encountered.

Out of the 123 tortoises sampled, 89% (109/123) were considered parasitized, while 11% (14/123) had no evidence of parasites at the time of sampling. For all 123 tortoises sampled, 80% (99/123) of tortoises were infected with the pinworm,* Tachygonetria*, 47% (58/123) of tortoises were infected with strongyles,* Chapiniella*, 6% (7/123) of tortoises were infected with* Augusticaecum*, 7% (9/123) were infected with trematode* Telorchis*, 10% (12/123) were infected with the protozoans* Eimeria*, 1% (1/123) were infected with* Cryptosporidium*, and 1% (1/123) were infected with amoebas. Parasite loads, approximated from egg counts, also varied for the 123 tortoises sampled. All endoparasites were found in low parasite loads.* Tachygonetria* and* Chapiniella* were the only parasite species also found in moderate and high loads. Similar trends were observed for the 112 tortoises used in the following analyses to determine differences in parasite prevalence (Table 1) and approximate loads (Table 2) among various* G. polyphemus* host and environmental characteristics.

Both male and female tortoises had six of the seven endoparasites identified; however* Cryptosporidium *was only found in one male, and amoebas were only found in one female. There were no significant differences found (*p* value <0.05) in the parasite prevalence or loads among sexes (57 females, 42 males).

The number of endoparasites differed in respect to size class, with the adults cumulatively harboring seven endoparasites (*Tachygonetria, Chapiniella, Augusticaecum, Telorchis, Eimeria, Cryptosporidium*, and amoebas) and subadults being infected with three endoparasites (*Tachygonetria, Chapiniella,* and* Eimeria*). However, no significant differences were seen (*p* value<0.05) in the parasite prevalence or loads among adults and subadults (104 adults, 8 subadults). Although not included in the analysis, four endoparasites were identified from juvenile tortoises (*Tachygonetria, Chapiniella, Augusticaecum,* and* Eimeria*).

The number of endoparasites varied between habitat types with only pinworm, cyathostomes, and ascarids present in all habitat types. Collectively, pine dominated habitats contained six endoparasites (*Tachygonetria, Chapiniella*,* Augusticaecum, Telorchis, Eimeria,* and* Cryptosporidium*), scrub dominated habitats contained six endoparasites (*Tachygonetria, Chapiniella*,* Augusticaecum, Telorchis, Eimeria,* and amoebas), and the single coastal strand habitat contained three endoparasites (*Tachygonetria, Chapiniella*, and* Augusticaecum*). No significant differences (*p* value <0.05) in parasite prevalence or loads were found among pine, scrub, and coastal strand habitats (31 pine, 61 scrub, and 20 coastal strand).

The number of endoparasites identified between sampling sites varied (Table 1), with only pinworm and cyathostomes present in all populations. Collectively, tortoises from BSP were found to have five endoparasites; FAUP had four endoparasites; PJP had six endoparasites; JDSP Scrub had three endoparasites; JDSP Pine had three endoparasites; and MD had three endoparasites. Parasite prevalence among sites differed (Table 1).* Chapiniella* prevalence was significantly lower in BSP compared to FAUP (*p* value <0.02438) and significantly lower in JDSP Pine compared to FAUP (*p* value <0.0155).* Telorchis* prevalence was significantly higher in BSP compared to FAUP (*p* value < 0.0243) and MD (*p* value <0.0216).* Eimeria* prevalence was significantly higher in BSP compared to JDSP Scrub (0.04885) and MD (*p* value <0.04918) and significantly higher in PJP compared to JDSP Scrub (*p* value <0.03224) and MD (*p* value <0.03604). Results for parasite loads among sites were also mixed (Table 2), with* Telorchis* and* Eimeria *showing significant differences in respect to site (*p* values <0.004, 0.010, respectively). Pairwise comparisons revealed that there was a significantly higher number of tortoises with low* Telorchis* loads at BSP when compared to FAUP, MD, JDSPS, and JDSPP (*p* values <0.001, 0.001, 0.004, and 0.002, respectively). There was also a significantly higher number of tortoises with low* Eimeria* loads at BSP when compared to JDSPP, JDSPS, and MD (*p* values <0.020, 0.009, and 0.010, respectively). Low* Eimeria* loads in tortoises at PJP were also significantly higher compared to JDSPP, JDSPS, and MD (*p* values <0.023, 0.012, and 0.013, respectively).

Regarding the three tortoises sampled twice, parasite presence and loads differed between sampling periods. Tortoise A (FAUP) had a moderate amount of* Tachygonetria* and low* Chapiniella *eggs present in the feces after the first sampling, and after the second sampling, only low* Tachygonetria* eggs were present. Tortoise B (FAUP) had low numbers of* Chapiniella *eggs present in the feces after the first sampling, and after the second sampling, it did not have any parasite eggs present. Tortoise C (BSP) displayed lower amounts of* Tachygonetria *eggs and* Eimeria* oocysts in the feces after the first sampling, and after the second sampling, it had high amounts of* Tachygonetria*, low* Chapiniella, *and low* Augusticaecum *eggs present.

## 4. Discussion and Conclusions

This study aimed to establish a gastrointestinal parasite baseline for South Florida gopher tortoise populations, while examining the potential influence various environmental and host factors have on parasite presence and approximate loads. This investigation of 123 tortoises found at least seven species of endoparasites in six genera and represents the first comprehensive gopher tortoise intestinal parasite study in the subtropical region of South Florida. These genera have been recorded in previous gopher tortoise surveys, which suggests that these endoparasites are part of the normal gastrointestinal flora of gopher tortoises across their range (temperate and subtropical). Three nematodes (*Tachygonetria, Chapiniella,* and* Augusticaecum*), one trematode (*Telorchis*), and at least three protozoans (*Eimeria, Cryptosporidium*, and amoebas) were among the parasites identified. Unfortunately, identification to species level was not possible since many nematode eggs are similar in appearance and adult worms are generally needed for a more detailed morphologic examination [33]. This examination is usually not accomplished without a necropsy [53].

*Tachygonetria* are a common genus of pinworm in the family Oxyuridae [54] and are routinely found in reptiles, tortoises included [32, 33, 55]. Oxyurids are generally considered nonpathogenic, although impactions with high parasite numbers have been reported [54, 56–58]. Parallel to earlier studies, pinworms were found to be the most prevalent parasite detected across the South Florida gopher tortoise populations. Interestingly, the genus of pinworm identified between studies varied;* Tachygonetria *spp. were identified in Louisiana [32], central Florida [30], and in this current South Florida study, while* Alaeuris *spp. were identified in Georgia [33] and central Florida [30].* Oxyuris *spp. were also identified in a previous central Florida study [30]. These differences suggest various species of pinworm naturally reside in gopher tortoises across their range.

*Chapiniella *spp*., *another endoparasite genus in the Strongylid family having been described previously in gopher tortoises [31, 33], was the second most prevalent endoparasite and found in the second highest loads across the South Florida gopher tortoise populations.* Chapiniella* prevalence rates were variable across sites; however, unlike the Georgia study, no sites lacked* Chapiniella* infections.

*Tachygonetria* and* Chapiniella* were the only two parasites identified throughout all sampling sites, sexes, age classes, and habitat types. Both* Tachygonetria *and* Chapiniella* are directly transmitted [59], and similar to McGuire's 2013 [33] study, the prevalence of* Tachygonetria* was not associated with* Chapiniella*.* Tachygonetria* and* Chapiniella* were also the only two parasites found in high, moderate, and low parasite loads. All other endoparasites were found in low parasite loads. These results, in conjunction with identification and prevalence data obtained from previous* G. polyphemus* endoparasite studies [28, 30–33], suggest pinworms and strongyles are relatively well established within the guts of most gopher tortoises across their range and naturally occur in higher numbers compared to other endoparasites detected in these tortoises. Unfortunately, the cause of these differences could not be determined from this study, as neither could their health effects upon the hosts.

The apicomplexan protist* Eimeria* is the most commonly known turtle and tortoise coccidian [55] and was the third most prevalent endoparasite found in this study. Since 2013, only two studies in Georgia have identified* Eimeria* in gopher tortoises [29, 33]. Generally,* Eimeria* is not associated with host disease, though high parasite burdens in younger individuals may cause clinical disease [33].

*Telorchis*, a genus of intestinal trematode [60] in the family Telorchiidae, was the fourth most prevalent endoparasite found in this study and the first report of a fluke in wild gopher tortoises. Flukes of this family comprise approximately 80 species and frequently reside in turtles, snakes, and salamanders as their definitive hosts around the world [61]. Although common in reptiles, little information exists on disease these flukes may cause [53].

*Augusticaecum*, a genus of ascarid, was the fifth most prevalent endoparasite found in South Florida gopher tortoises. This is the second report of an ascarid in gopher tortoises, the first being reported by McGuire et al. (2013) [33] in Georgia. These nematode roundworms may cause a variety of health problems, and with heavy infestations, fatal intestinal blockages can occur [62].

The protists* Cryptosporidium* and amoebas were the least prevalent endoparasites identified in this study. Both endoparasites have been previously identified in gopher tortoises [33, 63, 64]. Most of amoebiasis cases in reptiles are caused by* Entamoeba invadens*, yet, other species may cause disease [65, 66]. It is generally thought that most reptiles are “resistant to the disease”; however, clinical illness can progress. In fact, acute hepatic necrosis in a gopher tortoise has occurred [32, 33, 64, 66]. Although infrequent,* Cryptosporidium *spp. have been described in many species and can cause disease in tortoises [33, 67–70].

Our hypotheses predicting* G. polyphemus* intestinal parasite prevalence and loads to differ between sexes were not supported. Overall, males and females within this study seemed to harbor the same parasite species and in the same amounts. Diaz-Figueroa (2005) [32] and McGuire et al. (2013) [33] also found no differences in endoparasites between tortoise sexes.

Our hypotheses predicting endoparasite prevalence and loads to differ between size classes were also not supported, as no significant differences were found among adult and subadult tortoises. Similarly, in McGuire's 2013 [33] study (eight juveniles, 109 adults**)**, size class was also not found to be a significant factor in parasite prevalence [33]. Although these results suggest intestinal parasitism may be similar across tortoise size classes, and consequently age groups, definitive conclusions cannot be drawn since subadult and juvenile sample sizes were exceptionally low compared to adults. It also cannot be determined from this study as to when infection first occurs or the point at which these endoparasites become established within the gut.

Our hypotheses predicting* G. polyphemus* intestinal parasite prevalence and loads to differ between habitat types were not supported and suggest endoparasite prevalence and loads are relatively ubiquitous among the three South Florida habitat types examined in this study.

Our hypotheses predicting* G*.* polyphemus* intestinal parasitism to differ among varying environmental characteristics were only supported with respect to site. Observed site dissimilarities included lower parasite prevalence and loads in the larger, less dense, and regularly fire maintained pine and scrub habitats from JDSP than in the other pine and scrub habitats (BSP, FAUP, and PJP). The only exception was MD; contrary to what was originally anticipated for such a small, non-fire maintained, and highly dense site, MD also exhibited trends of having lesser intestinal parasite prevalence and loads than BSP, FAUP, and PJP. Trends seen at MD may be attributed to site specific characteristics including its largely open canopy that is more likely to allow UV radiation to degrade parasite eggs and larvae.

McGuire et al. (2013) [33] also described* G. polyphemus* intestinal parasite prevalence differences among sites and suggested that parasite transmission is likely dependent upon tortoise density. An increase in host density may lead to an increase in parasites and the probability of transmission [71]. This is especially true for those parasites that have direct life cycles, since the direct host will usually pass the infective stages via feces into the environment, and the next host may ingest them [53]. Consequently, the location and distribution of burrows may also influence transmission [33]. Density estimates for the sites used in this study relied on various survey methods and did not account for clustering of burrows/tortoises within individual sites. Therefore, conclusive evidence on whether density influences parasitism in wild South Florida populations was not attainable. A clearer understanding of host density on parasitism may be achieved if density could be quantified in a universal matter, accounting for the spatial distribution of burrows within sites.

McGuire et al. (2013) also attributed site differences to low sample sizes, possible variations in environmental characteristics, and land use among populations [33]. The same is likely true in this study, as various host and environmental characteristics that were not quantified have the potential to influence parasitism and disease. Interestingly, Diaz-Figueroa's 2005 study found no differences in parasite species between sites [32].

The seasonality of endoparasite shedding was evidenced by the three tortoises sampled twice; both parasite prevalence and approximate loads differed between sampling periods. Intestinal parasite eggs/oocysts are shed in feces sporadically and can lead to false negatives. Therefore, false negative results may not necessarily correlate to a lack of infection altogether. The same is true for the 11% (14/123) of tortoises found in this study to have no parasites at the time of sampling. Optimal results to determine true parasite prevalence and loads may be achieved if recurrent sampling from the same individual could be accomplished, although, with wild populations, capturing the same animal more than once is often difficult.

It should be noted that some years were more heavily sampled in certain seasons than other seasons, and some sites were completely sampled in just one year, while other sites were sampled over multiple years. It is generally known that parasites in the wild may fluctuate depending on the time of the year, the host-parasite relationship can be affected by season [72–75], and these changes can indirectly impact parasite transmission [75, 76]. Furthermore, it is speculated that the immunity of a host to disease can also fluctuate by season relative to alterations in reproduction, diet, strain, and light periods [75, 77]. Future studies should focus on sampling all sites throughout all four seasons and in the same years to more accurately measure sampling year and seasonal differences. Florida's wet and dry seasons should also be explored more in detail, since Florida does not typically experience the more drastic seasonal changes seen further north.

In conclusion, at least seven intestinal parasite species were identified in the South Florida gopher tortoise populations. Prior to this study, no comprehensive data on intestinal parasites has been published on* Gopherus polyphemus *from Florida, South Carolina, Alabama, or Mississippi [33]. We recommend future studies to include these states to create a baseline of gopher tortoise endoparasites in these locations. This work should include the identification of parasites to species, which generally requires the adult worms.

Long term monitoring should be continued in both urbanized and more natural populations to determine trends in parasitism as it relates to changes in land management, population structure, climate, and parasite life cycles. Confounding to this,* G. polyphemus* is a frequently translocated species [78] and understanding parasite distributions will be vital [33] in preserving these keystone species. Due to* G. polyphemus'* declining numbers and threatened status, further investigations will be of use to improve the management of this species and ensure its existence in the future.

## Figures and Tables

**Table 1:** Intestinal parasite eggs and oocysts in adult and subadult gopher tortoise (*Gopherus polyphemus*) fecal samples from six populations in South Florida.

Site	*n*	No. positive (%) *∗*						
		***Tachygonetria***	***Chapiniella***	***Augusticaecum***	***Telorchis***	***Eimeria***	***Cryptosporidium***	**Amoebas**
BSP	22	15(68)a	6(27)ac	1(5)a	6(27)a	5(22)ac	0a	0a
FAU	18	14(78)a	12(67)b	0a	0b	1(6)abc	0a	1(6)a
PJP	17	13(76)a	9(53)abc	1(6)a	1(6)ab	4(24)c	1(6)a	0a
JSPSS	21	17(81)a	12(57)abc	0a	1(5)ab	0b	0a	0a
JDSPP	14	13(93)a	3(21)ac	1(7)a	0ab	0abc	0a	0a
MD	20	17(85)a	10(50)abc	1(5)a	0b	0b	0a	0a
Total	112	89(79)	52(46)	4(4)	8(7)	10(9)	1(1)	1(1)

Different letters indicate significant differences in the prevalence between individual sites.

**Table 2:** Qualitative intestinal parasite loads approximated via egg counts in adult and subadult gopher tortoise (*Gopherus polyphemus*) fecal samples from six populations in South Florida.

Site	*n*	No. positive (%) *∗*										
		***Tachygonetria***			***Chapiniella***			***Augusticaecum***	***Telorchis***	***Eimeria***	***Cryptosporidium***	**Amoebas**
		L	M	H	L	M	H	L	L	L	L	L *∗*
BSP	22	9(41)	4(18)	2(9)	6(27)	0	0	1(5)	6(27)a	5(23)ac	0	0
FAU	18	4(22)	8(44)	2(11)	10(56)	1(6)	1(6)	0	0b	1(6)abc	0	1
PJP	17	5(29)	6(35)	2(12)	7(41)	2(12)	0	1(6)	1(6)ab	4(24)c	1(6)	0
JSPSS	21	11(52)	5(24)	1(5)	11(52)	1(5)	0	0	1(5)b	0b	0	0
JDSPP	14	4(30)	5(36)	4(30)	2(14)	1(7)	0	1(7)	0b	0b	0	0
MD	20	4(20)	9(45)	4(20)	8(40)	2(10)	0	1(5)	0b	0b	0	0
Total	112	37(33)	37(33)	15(13)	44(39)	7(6)	1(1)	4(4)	8(7)	10(9)	1(1)	1(1)

The letters L, M, and H represent the qualitative load values derived from approximated egg counts ((<1-1/LPF), (2-4/LPF), and (>=5/LPF)) provided by National Bio Vet Laboratories. Different letters indicate significant differences in the loads between individual sites.

## Data Availability

Parasite identification and load estimation results obtained by National Bio Vet Laboratories are provided in the Supplementary File (available here). Other data are available upon request from the corresponding author.

## Supplementary Materials

Supplementary Materials The supplementary file contains parasite identification and load estimation results obtained by National Bio Vet Laboratories, including the corresponding accession numbers.
